# Physiological and proteomic changes of *Castanopsis fissa* in response to drought stress

**DOI:** 10.1038/s41598-023-39235-x

**Published:** 2023-08-02

**Authors:** Chaonan Li, Sanxiong Chen, Yi Wang

**Affiliations:** College of Horticulture and Landscape Architecture, Zhongkai University of Agriculture and Engineering, Guangzhou, 510225 China

**Keywords:** Plant molecular biology, Plant stress responses

## Abstract

*Castanopsis fissa* is a native, broadleaf tree species in Guangdong with characteristics of barrenness and fast growth and is often used as a pioneer species for vegetation restoration with excellent ecological benefits. To explore the response of *C.fissa* to drought, this study investigated the drought tolerance mechanism of *C.fissa* using physiological and proteomic assessments. Using a potted continuous drought experimental method with normal water supply as a control, we measured photosynthetic parameters, antioxidant enzyme activities, and osmoregulatory substances of *C. fissa* in response to drought stress for 1 to 4 weeks, respectively. In addition, we used TMT quantitative proteomics to identify differentially expressed proteins (DEPs) between the drought-stress-treated *C. fissa* leaves and the control leaves. With the extension of drought stress time, the photosynthetic indexes and peroxidase (POD) activity of *C. fissa* leaves showed a decreasing trend. The malondialdehyde (MDA) content; superoxide Dismutase (SOD) and catalase (CAT) activities; and proline (Pro), soluble sugar (SS) and soluble protein (SP) contents showed an overall increasing trend, all of which reached significant differences at 4 w of stress. We identified 177 and 529 DEPs in the 2 and 4 weeks drought-stress leaves, respectively, in reference to the control leaves. These DEPs were closely related to physiological metabolic processes such as photosynthesis, energy and carbohydrate metabolism, stress response and defense, transcriptional regulation, and signal ion transduction. Drought stress mainly affects photosynthesis, carbohydrate metabolism, and protein synthesis and degradation in *C. fissa* leaves. At 2 weeks of stress, the expression of carbon metabolism, pyruvate metabolism and ribosome-related proteins was significantly changed, however, and at 4 weeks of stress, protein processing in the endoplasmic reticulum and spliceosome-related proteins were significantly increased in plant leaves. To alleviate the effect of water unavailability, the drought-stressed *C.fissa* leaves increased its oxidative protective enzyme system to eliminate excess reactive oxygen species (ROS) and also increased its Pro and SP contents to maintain the intracellular osmotic potential balance.

## Introduction

Water is one of the necessary elements for trees to survive, but currently, more than 1/3 of the world's land is in arid and semiarid regions, and other regions often experience different degrees of drought during the plant growing season. Drought has become one of the greatest environmental stresses for plants to complete their life cycle, so the study of drought resistance of forest trees and the mechanism of drought resistance is the basis of species selection for afforestation in arid areas.

Drought stress causes stomatal closure and decreased transpiration rates, a decline in the turgor pressure of plant cells, decreases in photosynthetic rate, leaf withering, and growth cessation^1^. Under adverse stress, plant survival is largely dependent on the response of many physiological and biochemical processes, and plants adapt to the external environment through cellular perception and transmission of signals from the external environment and regulation of gene expression to balance their own state^1,2^. In recent years, researchers have continued to explore the mechanisms by which plants respond to drought stress from various aspects and have made great progress. Studies have shown that the amount of reactive oxygen species (ROS) rise in plants during stress, causing the antioxidant enzyme systems (e.g., SOD, POD, CAT, etc.) in plants to initiate protective mechanisms to keep metabolism in balance^3^, while drought stress induces the production of soluble protein (SP), soluble sugar (SS) and free proline (Pro), which are important osmoregulatory substances in plants and have the functions of providing energy, increasing cytoplasmic concentration and enhancing the transmission of signal substances^3–5^. In the field of bioinformatics, Valdés et al. investigated the adaptive response mechanisms of two *Eucalyptus globulus* provenances to drought stress using proteomics. Their findings suggest that the drought-tolerant genotype has a more developed root system and can tolerate a higher degree of dryness^6^. The key metabolites (including proline, betaine, mannitol, etc.) of Thyme (*Thymus mongolicus*) that are significantly altered by drought stress and their pathways and modes of action were identified at the metabolic level by Parviz et al.^7^; Dong et al. identified a large number of cDNA single genes from tea oil camellia (*Camellia oleifera*) by transcriptome and further analyzed them to derive 20 candidate drought genes^8^. Protein profiling is an effective tool for discovering key molecular components of developmental or regulatory programs, and proteomic methods enable comparative analysis of protein abundance in tolerant and sensitive genotypes, which have greatly facilitated the study of drought stress responses in plant cells^6,9^. Proteins, as executors of gene functions, can be directly involved in plant responses to drought stress, and this response is reflected not only in direct changes in enzyme species and activities at the metabolic level but also in the structural and functional changes in plant cell membranes, cytoplasm, cytoskeleton, and many intracellular protein components through the regulation of transcription and translation levels. When plants are under stress, there is a close relationship between changes in their physiological indicators and the execution of protein functions. Changes in physiological indicators can be explained by changes in protein expression or function, and protein synthesis and degradation are also influenced to some extent by plant physiological changes.

*Castanopsis fissa* is an evergreen broad-leaved species of the cone genus *Castanopsis* in the family Fagaceae, which is widely distributed and is an excellent native species in Guangdong Province of China. This species has a well-developed root system, excellent soil-fixing power, and better soil and water conservation ability and has the characteristics of barren tolerance, strong sprouting power, abundant and easily decomposed dead leaves, etc. Therefore, *C. fissa* is not only a pioneer species for postdestruction sprouting forests but also a highly potential ecological public welfare forest tree species. Moreover, due to its beautiful shape, wide canopy and various colors, it has become an ideal tree for landscaping and ornamental purposes. However, there is a basic gap in knowledge in the reports on the drought resistance or drought tolerance genes of *C. fissa*. Based on the above details, in this study, 2-year-old *C. fissa* seedlings were used as the study material to investigate the plant physiological response under drought stress using a pot-based experiment. Furthermore, we compared and analyzed the differentially expressed proteins (DEPs) under different periods of drought stress by TMT quantitative labeling protein.

## Results

### Plant morphological changes

**Figure 1:**
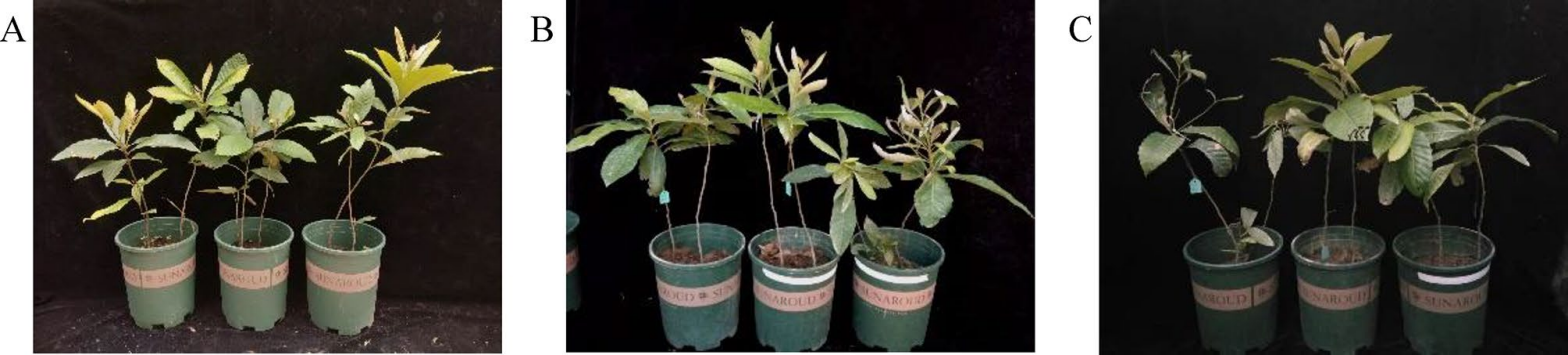
Effects of drought stress on plant growth status. (**A**) indicates control plants, (**B**) indicates plants under 2 weeks of drought stress, and (**C**) indicates plants under 4 weeks of drought stress.

When plants are under drought stress, the external manifestation of the plant mainly occurs on the leaves, which wilt due to water deficiency. As the degree of drought stress on the plant intensifies, the leaves gradually turn yellow, wilt and fall off, and the plant's vitality decreases. Under normal environmental conditions, the plants grow well with normal leaf color. With the increase of drought stress, at 2 weeks of drought stress, the plant grows well with only partial curling of new leaves; at 4 weeks of drought stress, the plant growth slows down, and the edges of the leaves curl and scorch (Fig. 1).

### Photosynthetic parameter changes

**Figure 2:**
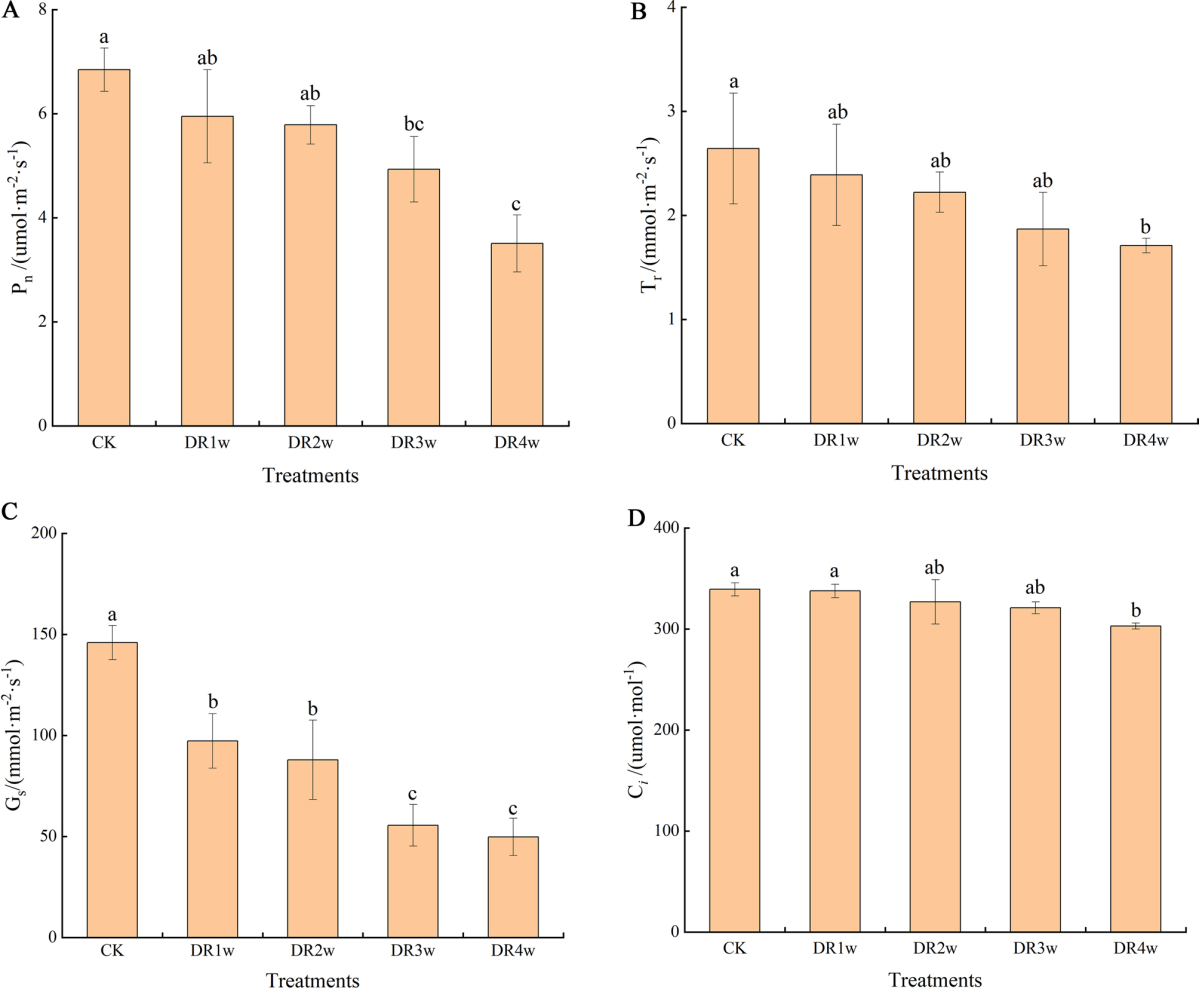
Effects of drought stress on plant photosynthesis. Different small letters indicate significant differences at *P* < 0.05; Same notes below. (A) net photosynthetic rate; (B) transpiration rate; (C) stomatic conductance; (D) intercellular CO_2_ concentration.

As shown in Fig. 2, the net photosynthetic rate(P_*n*_), transpiration rate(T_*r*_), stomatic conductance(G_*s*_), and intercellular CO_2_ concentration(C_*i*_) of *C. fissa* seedlings showed a decreasing trend with the prolongation of drought stress. Compared with CK, P_*n*_ showed no significant difference under drought stress for 1 to 2 weeks, but significant difference (*p* < 0.05) appeared at the third week, and the decline was 48.8% until 4 weeks of drought stress; T_*r*_ decreased steadily during the drought stress process until it showed a significant difference (*p* < 0.05) at the fourth week, with a total decrease of 35.3%; G_*s*_ significantly decreased after 1 week of drought stress and remained significantly different from the control group after 1 week. From 0 to 4 weeks, G_*s*_ decreased by 65.9%; C_*i*_ showed the smallest changes with increasing drought stress and at the end of the stress period, it only decreased by 10.7% compared to the control group. Overall, the decrease in Ci was smaller, while the decrease in Gs was larger in *C. fissa* with the prolongation of drought stress. For detailed photosynthetic parameter data, please see Table S1.

### Changes in physiological response

**Figure 3:**
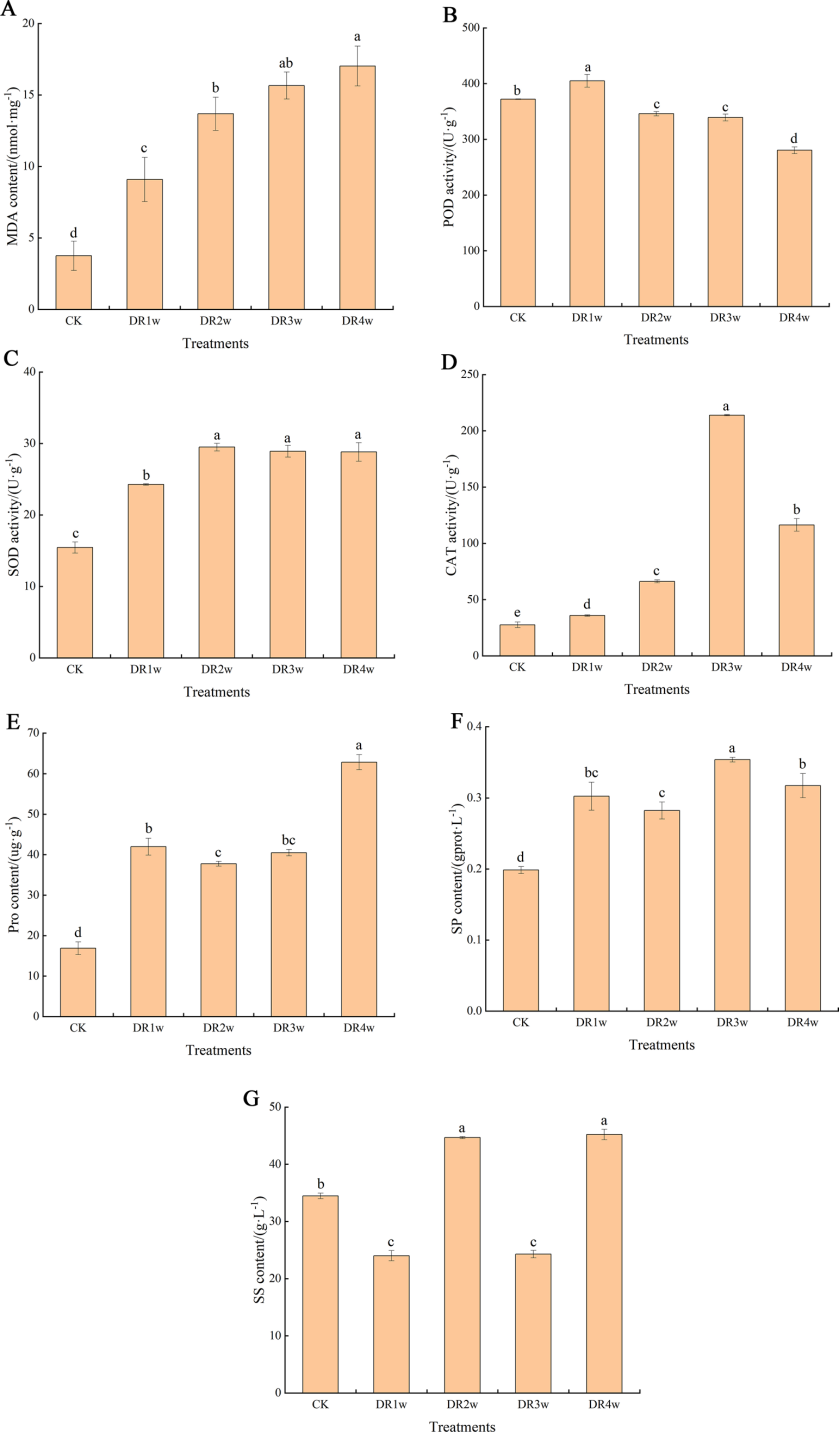
Effects of drought stress on plant physiological performance. (A) MDA contents; (B) POD activity; (C) SOD activity; (D) CAT activity; (E) Pro contents; (F) SP contents; (G) SS contents.

MDA is one of the important indicators to determine the membrane damage caused by drought effects on plants. As shown in Fig. 3A, the stress-induced MDA content displayed a positive correlation with the duration of drought stress. These data demonstrated a significant increase in MDA (*P*-values < 0.05) when *C. fissa* seedlings were treated with drought stress for 4 weeks. The three enzymes SOD, POD and CAT synergize to resist the damage caused by the adverse environment when plants are exposed to adversity stress^10^. With the extension of stress time, the levels of POD, SOD and CAT in plants increased and then decreased, but the time points at which the turning points occurred were different (Fig. 3B–D). The POD activity increased significantly in the first week of drought stress but started to decrease consistently after 2 weeks of treatment and was significantly lower than that in the CK group (*P*-values < 0.05), with a 24.7% decrease in POD activity between 0 and 4 weeks. The SOD and CAT activities persistently and significantly increased at 1 ~ 2 weeks of drought stress; however, divergence occurred at DR3w, with a slight decrease in SOD activity but a rapid increase in CAT activity. During the period from 0 to 4 weeks, SOD and CAT activities increased by 46.4% and 76.3%, respectively, compared with the control. Drought stress causes a series of problems, such as water loss, reduced cytosol concentration and cell expansion imbalance in plants. Pro, SP and SS are important osmoregulatory substances in plants. From Fig. 3E,F, with the increase in treatment days, the trends of Pro and SP contents in *C. fissa* leaves were similar, with an overall increasing trend and significantly higher values than CK values at each stage. Compared to those in the CK group, Pro and SP contents increased by 73.2% and 37.2%, respectively, after 4 weeks of drought stress. As shown in Fig. 3G, the SS content showed a significant fluctuating change with a further increase in drought stress, and the maximum content was observed at week 4. Please see Table S1 for detailed physiological data.

### Results of protein identification

The *C. fissa* leaves of CK, DR2w and DR4w were taken for protein identification. After each sample was detected by LC‒MS/MS and searched for libraries, a total of 366,195 secondary mass spectra were obtained. Among these spectra, we identified a total of 61,495 spectra corresponding to known spectra, 26,898 peptides, 20,545 unique peptides, accounting for 76.4% of the total identified peptides, and a total of 6626 proteins (Table 2). The qualitative and quantitative results of the proteins are shown in Table 1 and Table S2.

**Table 1:** Basic protein identification information.

Database	MS/MS	PSMs	Peptides	Unique peptides	Protein groups
UniProt taxonomy	366,195	61,495	26,898	20,545	6626

Database: the name of the database species used; MS/MS: total secondary spectra; PSM: peptide spectrum match, the number of peptides matched to secondary spectra; Peptides: total number of different peptides identified; Unique peptides: total number of unique peptides identified; Protein groups: total number of proteins identified.

### Screening of DEPs

**Figure 4:**
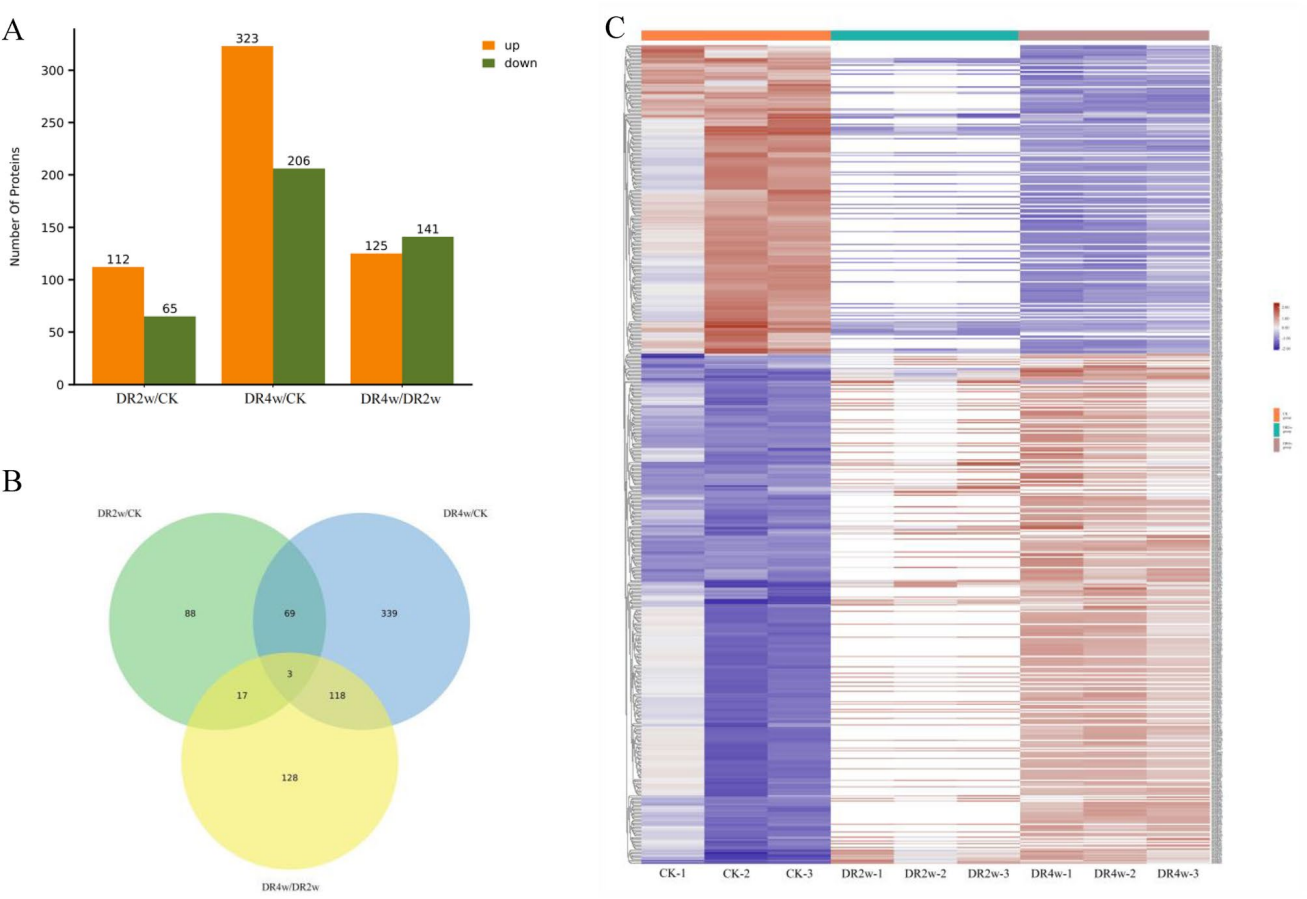
Differential protein statistics in DR2w/CK and DR4w/CK comparison groups. (1) Panel (**A**) shows the statistical table of DEPs; panel (**B**) shows the DEPs Venn diagram; panel (**C**) shows the heatmap of clustering analysis of differential comparison groups. (2) Panel (**C**), clustering heatmap, red indicates high expression protein, blue indicates low expression protein, and each row indicates the expression of each protein in different groups.

The CK group, DR2w and DR4w were compared in a two-by-two comparison (i.e., DR2w/CK, DR4w/CK, DR4w/DR2w), fold change = 1.5-fold and p-values < 0.05 were set for screening of DEPs. As shown in Fig. 4A and Table S3, compared with the CK group, there were 177 DEPs in *C. fissa* leaves of DR2w, of which 112 DEPs were upregulated and 65DEPs were downregulated. There were 529 DEPs in the leaves of DR4w, of which 323 DEPs were upregulated and 206 DEPs were downregulated. There were 266 DEPs in DR4w leaves compared to DR2w leaves, of which 125 DEPs were up-regulated and 141 were downregulated. In the comparison groups of DR2w/CK and DR4w/CK, a total of 72 coacting DEPs were found, and these proteins may occupy a leading position in the resistance of *C. fissa* seedlings to drought stress (Fig. 4B). The expression of each protein can be clearly seen in Fig. 4C and and Table S3.

### Gene Ontology (GO) enrichment analysis of DEPs under drought stress

**Figure 5:**
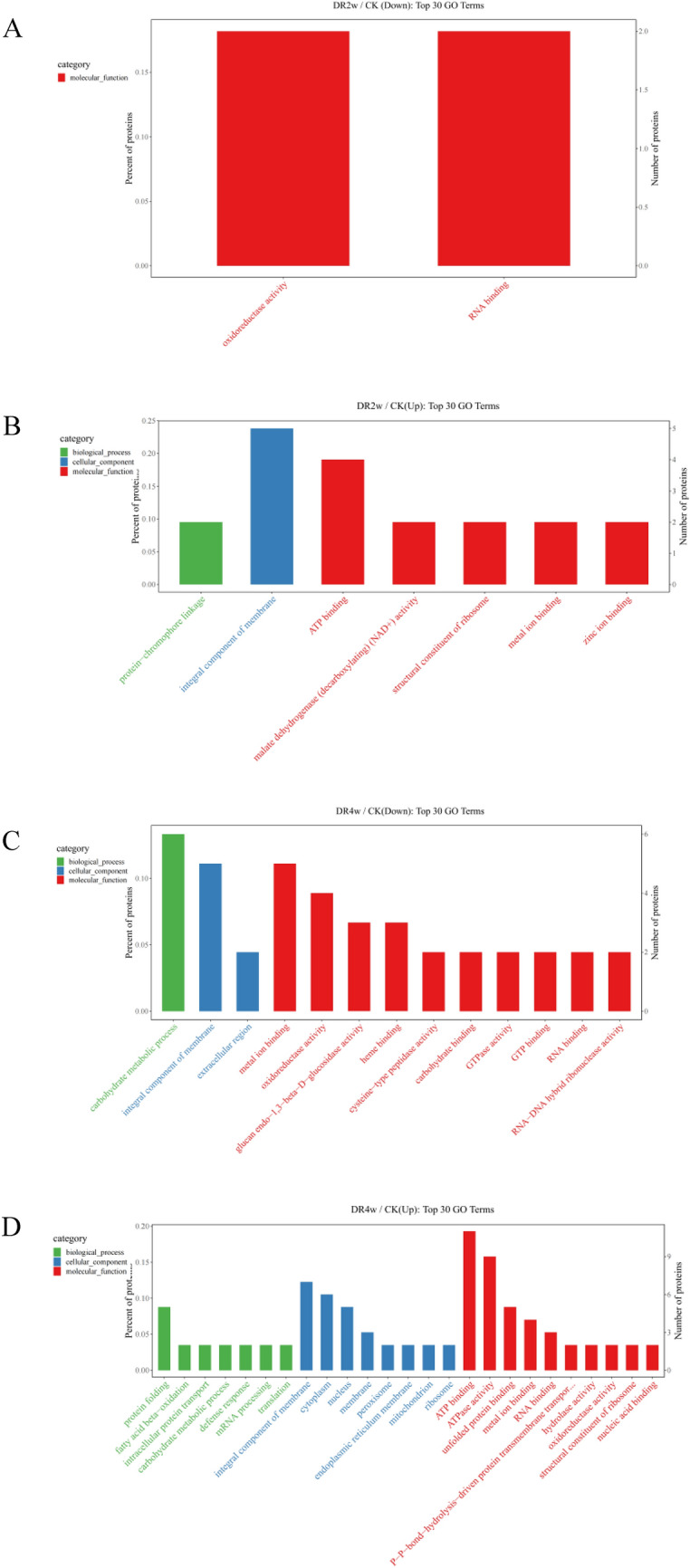
GO enrichment analysis of DEPs in DR2w/CK and DR4w/CK. (1) (**A**) indicates the DEPs downregulated by DR2w/CK; (**B**) indicates the DEPs upregulated by DR2w/CK; (**C**) indicates the DEPs downregulated by DR4w/CK; (**D**) indicates the DEPs upregulated by DR4w/CK. (2) The x-coordinate in the graph is the name of GO entry, and the y-coordinate is the number of proteins in the corresponding entry and their percentage.

GO annotations can classify the expression of proteins in three ways: biological process, molecular function and cellular component^8^. In this study, we used all the proteins identified as a background list, and the DEPs of *C. fissa* leaves under different drought treatment times were used as a candidate list for GO function enrichment analysis. The bar diagram of GO enrichment analysis top 30 (Screening of GO terms corresponding to the number of DEPs greater than 1 in the three classifications, and then 10 entries each sorted by the -log10P-value corresponding to each entry from largest to smallest) is shown in Fig. 5.

Figure 5A,B shows the comparison of DR2w with CK. The downregulated DEPs were all significantly enriched in the ‘molecular function’ GO terms ‘oxidoreductase activity’ (GO:0016491) and ‘RNA binding’ (GO:0003723). For the upregulated DEPs, the most enriched term in the molecular function category was 'ATP binding' (GO:0005524), followed by the ‘cellular component’ GO term ‘integral component of membrane’ (GO:0,016,021), meaning that the proteins involved in these processes may play a vital role in drought sensing. Figure 5C,D shows the GO enrichment results of DEPs in ‘DR4w/CK’, and the DEPs that were significantly enriched in GO terms (DEPs ≥ 4) among the downregulated DEPs included ‘carbohydrate metabolic processes’ (GO:0005975) involved in biological processes, ‘integral components of membranes’ (GO:0016021) participating in cellular components, ‘metal ion binding’ (GO:0046872) and ‘doxidoreductase activity’ (GO:0016491) involved in molecular function. For the upregulated DEPs, the significantly enriched molecular function GO terms included ‘ATP binding’ (GO:0005524) and ‘ATPase activity’ (GO:0016887). In addition, the more upregulated DEPs (DEPs ≥ 4) were also enriched in cellular component terms, including ‘integral component of membrane’ (GO:0016021), ‘cytoplasm’ (GO:0005737), and ‘nucleus’ (GO:0005634), and biological process terms, including ‘protein folding’ (GO:0006457).

### KEGG enrichment analysis of DEPs under drought stress

KEGG is the main public database for systematic analysis of metabolic pathways of proteins in cells^11–14^. In this study, pathway analysis of DEPs was performed using the KEGG database to further understand the enrichment pathways of DEPs in *C. fissa* leaves under different degrees of drought stress. According to the KEGG pathway obtained from this analysis, the altered expression of DEPs mainly involves cellular processes, environmental information processing, genetic information processing and metabolism.

**Figure 6:**
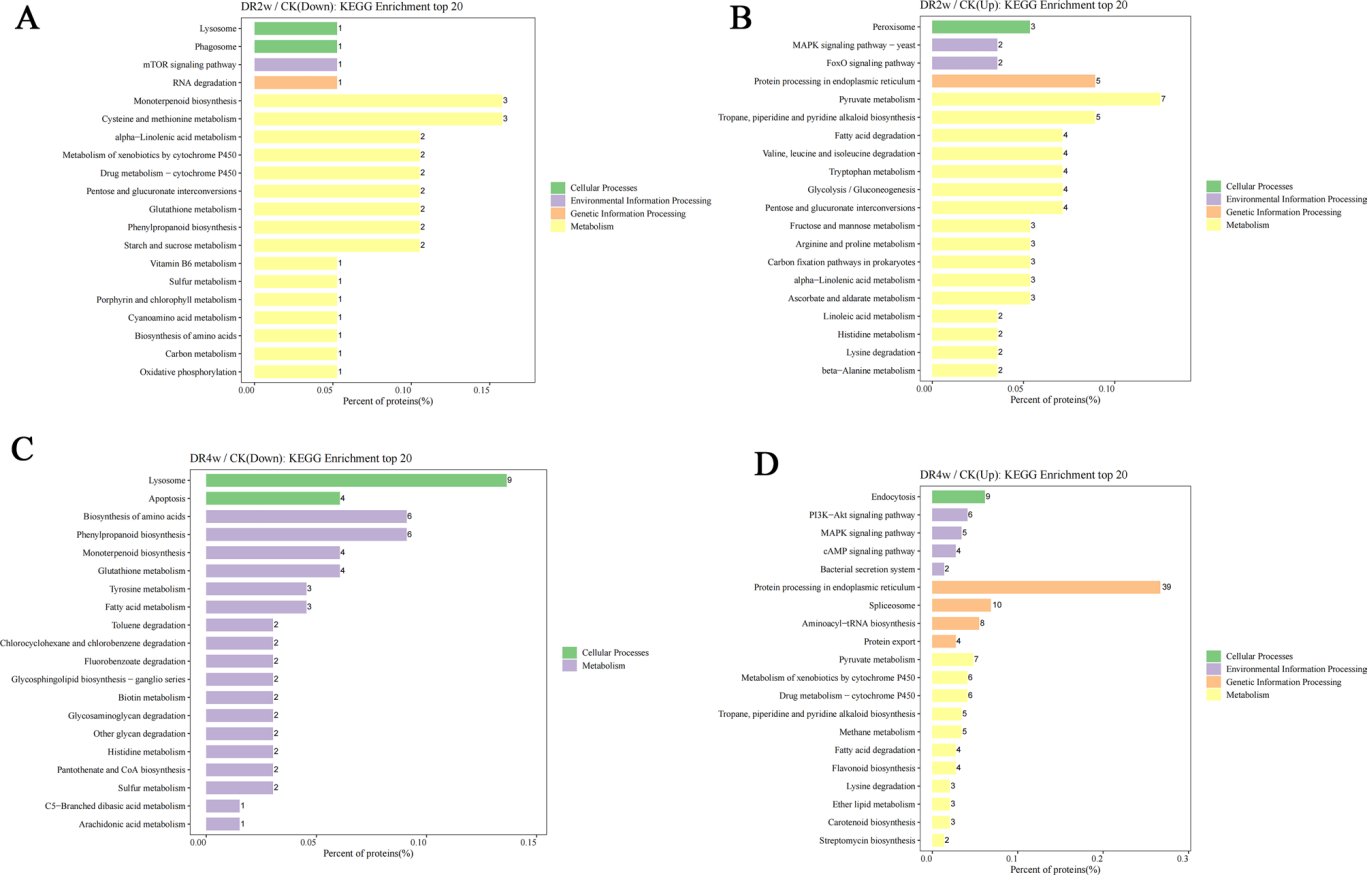
KEGG enrichment analysis of DEPs in DR2w/CK and DR4w/CK. (1) (**A**) indicates the DEPs downregulated by DR2w/CK; (**B**) indicates the DEPs upregulated by DR2w/CK; (**C**) indicates the DEPs downregulated by DR4w/CK; (**D**) indicates the DEPs upregulated by DR4w/CK. (2) The x-axis Percent of proteins is the percentage of protein expression, and the y-axis is the pathway information of the top 20 DEPs.

Figure 6A,B shows the KEGG metabolic pathways for the DEPs in “DR2w/CK”..The DEPs observed in the enriched KEGG pathway were mainly focused on metabolic processes. The downregulated DEPs were predominantly mapped onto pathways such as ‘monoterpenoid biosynthesis’, ‘cysteine and methionine metabolism’, and ‘phenylpropanoid biosynthesis’ in metabolism. Among the upregulated DEPs, most were associated with ‘tropane, piperidine and pyridine biosynthesis,’ ‘protein processing in the endoplasmic reticulum’ in metabolism, and ‘pyruvate metabolism’ in genetic information processing. The DEP expression and number changes indicate that the plant is undergoing an active and complex response to counteract external adverse environmental changes.

With the further increase in drought stress, the number of DEPs enriched by the KEGG pathways in “DR4w/CK” increased substantially compared with “DR2w/CK”. As shown in Fig. 6C,D, the downregulated DEPs were essentially distributed in the classification of cellular processes and metabolism, including ‘lysosome’ and ‘apoptosis’ in cellular processes and ‘biosynthesis of amino acids,’ ‘phenylpropanoid biosynthesis,’ ‘monoterpenoid biosynthesis,’ and ‘glutathione metabolism’ in KEGG metabolism pathways. For the upregulated DEPs in the ‘DR4w/CK’ comparison, the most significantly enriched ‘genetic information processing’ KEGG category was ‘protein processing in endoplasmic reticulum’ pathway, followed by the same category ‘spliceosome’ and ‘aminoacyl − tRNA biosynthesis’ pathway. The KEGG pathway is also enriched with many DEPs, including but not limited to ‘endocytosis’ in cellular processes, ‘PI3K − Akt signaling pathway’ and ‘MAPK signaling pathway’ in environmental information processes, and ‘pyruvate metabolism,’ ‘metabolism of xenobiotics by cytochrome P450,’ and ‘drug metabolism − cytochrome P450’ in metabolism.

### Identification of protein–protein interaction (PPI) networks among DEPs

**Figure 7:**
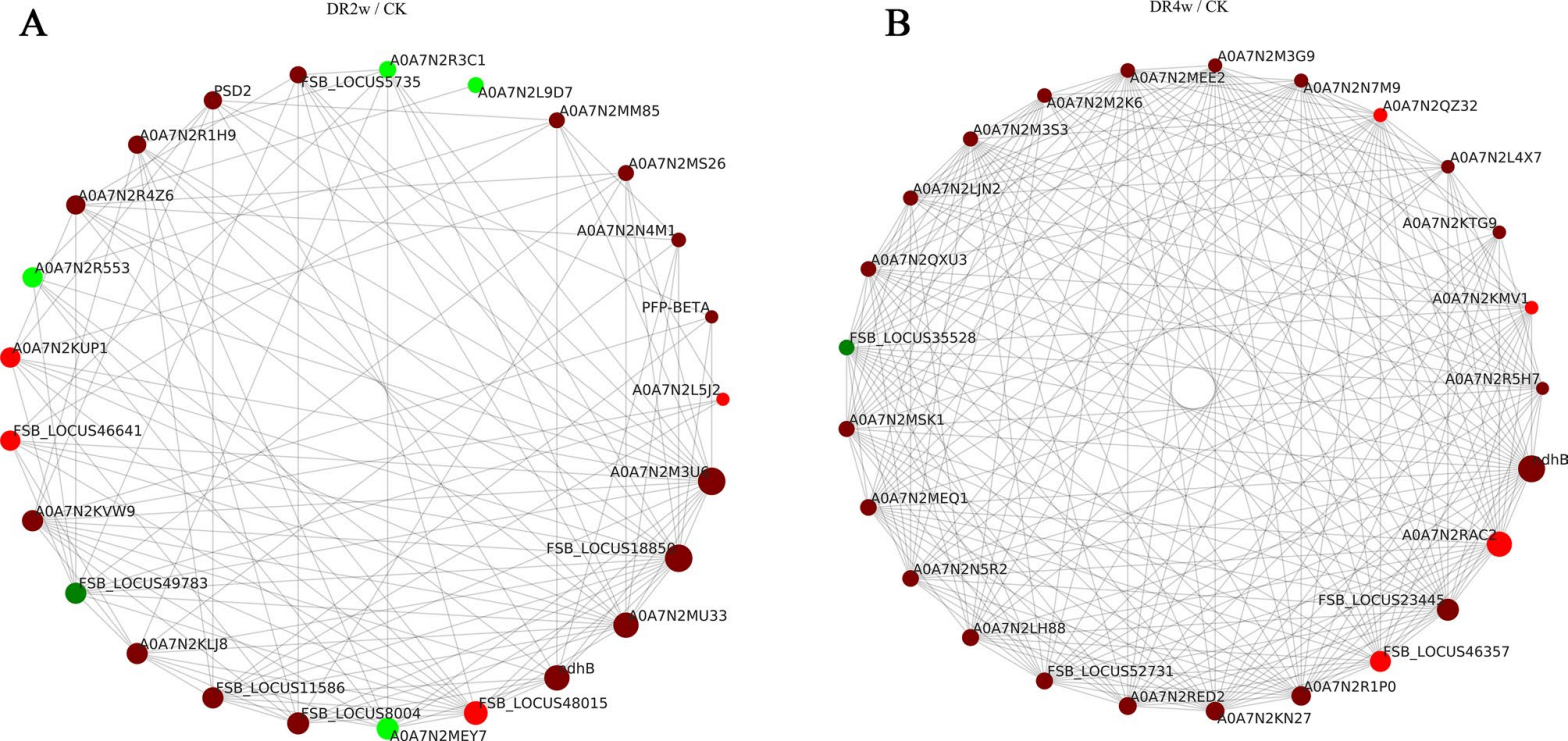
PPI analysis of DEPs in DR2w/CK (A) and DR4w/CK (B). The circles in the figure represent DEPs, red represents upregulated DEPs, and green represents downregulated DEPs; the size of the circles represents the level of connectivity, with larger circles indicating higher connectivity.

Protein expression in organisms is a complex network system, and these proteins have cooperative or inhibitory effects with each other^15^. In this study, we analyzed the DEPs by using the String (https://string-db.org/) database to obtain the interactions of DEPs under drought stress and selected the top 25 proteins in terms of connectivity for interaction network mapping. As shown in Fig. 7A, in the comparison of DR2w and CK, a total of 46 DEPs were involved in the protein interaction network under drought stress, among which PKS_ER domain-containing protein (FSB_LOCUS18850) had 20 reciprocal nodes, NAD(P)H-quinone oxidoreductase subunit 2, chloroplastic (ndhB) and aldehyde dehydrogenase (NAD( +)) (A0A7N2MU33) each had 17 nodes, and malic enzyme (A0A7N2KVW9) and cysteine synthase (A0A7N2R3C1) had 12 and 8 node connections, respectively. As shown in Fig. 7B, in the comparison of DR4w and CK, a total of 165 DEPs were involved in the network of protein interactions under drought stress, among which NAD(P)H-quinone oxidoreductase subunit 2, chloroplastic (ndhB), HATPase_c domain-containing protein (A0A7N2RAC2), phosphopyruvate hydratase (FSB_LOCUS23445), CCT-epsilon (A0A7N2R1P0) and CCT-eta (A0A7N2KN27) had more than 50 association nodes, and the top 25 DEPs all had more than 38 node connections.

### Screening of key DEPs in leaves of *C. fissa* seedlings under drought stress.

Based on the results of GO and KEGG enrichment analysis, the significantly different entries or pathways in the DR2w/CK and DR4w/CK comparison groups were analyzed, and the DEPs with more participating entries or pathways in the comparison groups were selected, while focusing on the DEPs that continued to play a role in both comparison groups. Then, combined with the regulatory network of action between proteins, the repeated proteins with connection points greater than 1 in the comparison groups were selected, and 125 DEPs (including 77 upregulated and 48 downregulated DEPs) were further screened. Under drought stress treatment, DEPs in the leaves of *C. fissa* seedlings were mostly involved in carbon metabolism (A0A2N9FA98, A0A2N9G1Y6, A0A7N2R3C1, etc.), Carbon fixation in photosynthetic organisms (A0A2N9J540, A0A2N9G1Y6, A0A2N9FA98, etc.), and protein processing in the endoplasmic reticulum (A0A7N2KYG4, A0A7N2LNX8, A0A7N2MIZ2). In addition, some of the proteins were also identified as having a certain number of interaction nodes but were not described (A0A2N9ESS5, A0A2N9IU54, etc.), and these proteins will be further investigated in the future. A portion of the key proteins are shown in Table 2. Based on the metabolic pathways of KEGG, the known proteins can be roughly classified into the following five categories: photosynthesis-related proteins, energy metabolism-related proteins, carbohydrate metabolism-related proteins, antioxidant-related proteins and signal transduction-related proteins.

**Table 2:** A portion of key proteins in the leaves of *C. fissa* seedlings under drought stress.

Accession	Gene name	P-value		FC		Up/Down	Protein name
		DR2w/CK	DR4w/CK	DR2w/CK	DR4w/CK		
Photosynthesis-related proteins							
A0A1N7TDW7	ndhB	0.03794	0.016001	1.66650	1.71519	Up	NAD(P)H-quinone oxidoreductase subunit 2, chloroplastic
A0A2N9FA98	FSB_LOCUS11586	0.01642	–	1.55335	–	Up	Kinesin motor domain-containing protein
A0A2N9G1Y6	FSB_LOCUS24679	0.04552	–	1.61723	–	Up	Malic enzyme
A0A2N9J540	FSB_LOCUS59485	0.03968	–	1.76395	–	Up	Phosphoenolpyruvate carboxylase
A0A6M4AXX4	psaB	0.04191	–	2.08671	–	Up	Photosystem I P700 chlorophyll a apoprotein A2
A0A2N9G789	FSB_LOCUS23162	–	0.01794	-	2.02187	Up	Protein translocase subunit SecA
A0A2N9I7X5	FSB_LOCUS47833	–	0.03315	–	2.03929	Up	CCT-epsilon
Energy metabolism-related proteins							
A0A2N9E520	FSB_LOCUS1929	0.00280	0.00117	0.30849	0.66375	Down	Beta-N-acetylhexosaminidase
A0A2N9EQL5	FSB_LOCUS5014	0.00280	0.00242	0.30849	0.51909	Down	Fe2OG dioxygenase domain-containing protein
A0A7N2L1J3	PSD2	0.04299	0.04746	1.78535	1.76010	Up	Phosphatidylserine decarboxylase proenzyme 2
A0A2N9FGN5	NBP35	–	0.02884	–	1.70103	Up	Cytosolic Fe-S cluster assembly factor NBP35
A0A2N9H7A0	FSB_LOCUS35403	–	0.03559	–	1.80323	Up	3-hydroxyacyl-CoA dehydrogenase
A0A2N9I8Z4	FSB_LOCUS48402	–	0.03070	–	1.79089	Up	Lactase
Carbohydrate metabolism-related proteins							
A0A2N9GKC7	FSB_LOCUS27633	0.01044	0.02278	1.81011	1.60599	Up	MFS domain-containing protein
A0A2N9IDB7	FSB_LOCUS49783	0.00254	0.00136	0.52770	0.58498	Down	Short-chain dehydrogenase/reductase
A0A2N9FUP9	FSB_LOCUS18850	0.00825	–	1.54735	–	Up	PKS_ER domain-containing protein
A0A2N9ETX0	FSB_LOCUS6013	0.00935	–	0.60167	–	Down	Pectinesterase
A0A2N9EP59	FSB_LOCUS4445	–	0.00291	–	0.43733	Down	Glucose-6-phosphate 1-epimerase
A0A2N9ELK1	FSB_LOCUS3547	–	0.00646	–	0.59209	Down	Beta-galactosidase
A0A2N9ES47	FSB_LOCUS5475	–	0.02429	–	0.62240	Down	(1- > 3)-beta-glucan endohydrolase
A0A2N9H8L4	FSB_LOCUS35806	–	0.00385	–	0.47976	Down	(1- > 3)-beta-glucan endohydrolase
Antioxidant-related proteins							
A0A2N9I211	FSB_LOCUS46574	0.01463	0.00222	2.79614	1.99164	Up	Endoplasmic reticulum transmembrane protein
A0A2N9IBX7	FSB_LOCUS50114	0.00064	0.00428	0.63442	0.65682	Down	Thioredoxin domain-containing protein
A0A2N9HF74	FSB_LOCUS38203	0.014837	–	1.68828	–	Up	Catalase
A0A2N9IM77	FSB_LOCUS53864	–	0.00077	–	1.72111	Up	Cir_N domain-containing protein
Signal transduction-related proteins							
A0A2N9I7S6	FSB_LOCUS48015	0.03900	0.03496	1.91155	1.85593	Up	Acyl-coenzyme A oxidase
A0A2N9ISF4	FSB_LOCUS54833	–	0.01452	–	2.76392	Up	HATPase_c domain-containing protein
A0A2N9IL82	FSB_LOCUS52731	–	0.04311	–	1.53012	Up	T-complex protein 1 subunit gamma
A0A2N9GX07	FSB_LOCUS32124	–	0.00947	–	0.62663	Down	Cysteine protease RD19A
A0A2N9HDZ3	FSB_LOCUS37676	–	0.01738	–	0.65682	Down	Thiol protease aleurain-like

“–” indicates that the protein does not comply with the differential screening condition of foldchange = 1.5-fold and p-value < 0.05 at this stage.

## Discussion

### Response of physiological indicators of *C. fissa* leaves under drought stress

Photosynthetically active leaves are part of the plant that is seriously affected by water limitation, which leads to reduced leaf emergence/expansion and shrinkage/curling^16^. Therefore, photosynthetic response-related data are often used as an important indicator of drought resistance. Generally, the factors affecting plant photosynthesis are classified into stomatal and nonstomatal factors. The former means that under water deficit conditions, plant leaf stomata are closed, and CO_2_ entry into the leaves is obstructed, leading to a decrease in the photosynthetic rate; thus, photosynthetic parameters at this stage show a decrease in C_*i*_ and P_*n*_. The latter is under heavy drought stress, where the photosynthetic structure of plant leaves is disrupted, the assimilation of carbon is weakened, C_*i*_ increases, P_*n*_ decreases, and both Tr and G_*s*_ are reduced at this time^17^. In this study, we found that with the prolongation of drought stress, P_*n*_, T_*r*_ and G_*s*_ showed a decreasing trend, and C_*i*_ continued to decrease, indicating that the photosynthetic efficiency of *C. fissa* leaves was unaffected by nonstomatal restriction during the drought experiment, which also shows that *C. fissa* has some drought resistance.

When plants are exposed to adverse stress, the accumulation of Reactive oxygen species (ROS) in plants usually causes membrane lipid peroxidation, thereby detrimental to plant growth. MDA, a product of lipid peroxidation, reflects the degree of membrane lipid peroxidation^1,18^. In this study, the persistent and significant increase in MDA content indicated that *C. fissa* was subjected to severe membrane damage during stress. Plants have evolved a variety of ROS scavenging strategies to eliminate the excess ROS produced by initiating antioxidant enzyme activities^19^. The antioxidant enzyme system mainly consists of SOD, POD and CAT, and all three act synergistically to protect plants from drought stress. SOD disproportionates O^2-^, CAT breaks down the H_2_O_2_ produced during disproportionation, and POD scavenges harmful free radicals and catalyzes the breakdown of substances to reduce the damage caused by ROS to plant cells^18,20^. In this study, the CAT and SOD activities of *C. fissa* showed a tendency to increase and then decrease with drought stress, while the POD activity showed an overall decreasing trend. According to the results, the antioxidant enzyme systems in *C. fissa* leaves mainly include CAT and SOD, which play a role in resisting drought and scavenging ROS in the drought experiment. To further elaborate, SOD reached its peak value at 2 weeks of drought stress, while CAT reached its peak value at 3 weeks of stress, indicating that SOD played a role earlier than CAT in the process of clearing ROS. Osmoregulation is an important mechanism for plants to respond to drought conditions, among which Pro, SP and SS are significant substances in the osmoregulation of plant leaves^21^. The Pro, SS and SP contents in *C. fissa* leaves demonstrated an increasing trend overall with the extension of stress. The Pro content increased exponentially and peaked at the 4th week of drought stress, as did the SS content, while the SP content peaked at the 3rd week of stress. When plants are suffering from drought stress, water deficit increases the intracellular osmotic potential, at which time the Pro content increases dramatically by increasing the cytoplasmic concentration to reduce the osmotic potential, thus maintaining the osmotic balance of the cell, while SS and SP increase the water absorption and retention capacity of plant leaves by reducing the cellular water potential^17^.

### Functional analysis of DEPs in *C. fissa* leaves under drought stress

#### Photosynthesis-related proteins

Photosynthesis mainly consists of two stages: light reaction and dark reaction, including important reaction steps such as light absorption, photosynthetic phosphorylation, and carbon assimilation^6^. In this study, 8 DEPs identified in leaves under 2 weeks of drought stress, relative to the control, were found to be involved in the regulation of photosynthesis. Among these proteins, only uroporphyrinogen decarboxylase (A0A7N2MEY7) showed downregulation during stress, indicating that the chlorophyll content showed a decreasing trend at this time, which had an impact on the photosynthetic rate. The photochemical reactions under photosynthesis include photosystem I and photosystem II, which are two multisubunit protein complexes embedded in the vesicle-like membrane, both as photosynthetic pigments and electron transporters^22^. The photosystem Ip700 protein (A0A6M4AXX4) and NAD(P)H-quinone oxidoreductase subunit2 (A0A1N7TDW7) are involved in the photoreactive phase, among which the photosystem Ip700 protein is engaged in the primary reaction phase of photosynthesis and participates in the cyclic electron transfer chain while transferring electrons from plastid blue pigment to iron redox proteins, generating ATP in the process^23^; NAD(P)H-quinone oxidoreductase subunit 2 catalyzes quinones and their derivatives to undergo reduction reactions, preventing the formation of oxidation products such as free radicals, which can prevent damage to cells^23^. Five DEPs were identified to be involved in the process of photosynthetic carbon fixation, including phosphoenolpyruvate carboxylase (PEPC) (A0A2N9J540, A0A7N2R1H9), kinesin motor domain-containing protein (A0A2N9FA98) and malic enzyme (A0A2N9G1Y6, A0A7N2KVW9). PEPC, a cytoplasmic enzyme, is involved in catalytic reactions, which are the main steps in CO_2_ assimilation and fixation related to C4 metabolism and saprophytic acid metabolism in plant photosynthesis, and is capable of participating in other nonphotosynthetic processes to regulate plant tolerance to stresses of adversity^24,25^. Malic enzyme's main function is to supplement the carbon demand of photosynthesis under drought stress by catalyzing the reaction to generate CO_2_^26,27^. Kinesin motor domain-containing protein is also involved in the carbon fixation part of photosynthesis. The upregulated expression of these photosynthesis-related proteins indicates that the photosynthetic process within the *C. fissa* leaves develops a positive response strategy to drought stress during this phase.

Compared to leaves stressed for 2 weeks, the additional prolycopene isomerase (A0A7N2M435, A0A2N9FN15) showed upregulated expression and participated in the carotenoid biosynthesis in leaves subjected to 4 weeks of drought stress. By investigating the protective mechanisms of photosynthetic chromophores and nonphotosynthetic chromophores against photosynthesis in *Phillyrea latifolia* under drought conditions, Antonella Gori et al. strongly demonstrated that carotenoids have antioxidant effects in leaves of plants suffering from the most severe drought stress, preventing lipid membrane peroxidation while protecting chlorophyll from photooxidative damage^28^. Therefore, in our study, the biosynthesis of carotenoids may have similar functions to that of *P. latifolia*. Ferredoxin-NADP reductase (A0A7N2LT68) and ribose-5-phosphate isomerase (A0A7N2LRE5) showed downregulated expression, with the former mainly participating in the light reaction steps in photosynthesis and acting as a transmitter of hydrogen bodies in enzymatic reactions, while the latter was involved in the dark reaction steps and was an essential enzyme in the pentose phosphate pathway and the Calvin cycle. This result indicates that at 4 weeks of drought, the light response and carbon sequestration capacity of *C. fissa* were severely affected, resulting in a decrease in photosynthetic capacity, and this trend is also consistent with the results of photosynthetic rate indicators in the previous physiological experiments.

### Energy metabolism-related proteins

Metabolism is a fundamental feature of the organism's life activity, and energy metabolism is an important component of metabolism. Even under conditions of adversity, energy is constantly being broken down or synthesized in the plant life cycle. The original water balance and osmotic pressure balance in plants are injured when under drought stress, for which plants need to initiate a series of defensive measures to maintain normal growth, and all these processes require energy consumption^29^.

In this study, 21 DEPs identified in the drought-stressed leaves for two weeks, relative to the control, were found to be involved in the regulation of energy metabolism (15 upregulated and 6 down-regulated). Under drought stress, plant cell amino acid metabolism can rapidly synthesize affinity osmotic substances as a way to regulate cellular osmotic potential and maintain normal cell growth and development. Amino acids are capable of producing ATP to provide energy for plants in several ways, including catabolism to α-keto acids through deamination, conversion to sugars or fats, and oxidation through the tricarboxylic acid cycle to form CO_2_ and H_2_O^30^. Aldedh domain-containing protein (A0A7N2LAP4) and aldehyde dehydrogenase (A0A7N2MU33) are involved not only in the metabolism of many amino acids but also in fatty acid degradation and glycolysis. The diversity of response substances of both indicates the importance in the drought resistance process, and their upregulated expression also shows that photosynthesis is hindered and carbohydrate synthesis is reduced under drought conditions; simultaneously, the internal metabolism of the plant is accelerated and requires continuous consumption of organic substances to supply energy to the plant.

After 4 weeks of drought stress treatment, proteins related to energy metabolism were significantly increased compared to 2 weeks of stress, with a total of 46 DEPs exhibiting changes (29 upregulated and 17 downregulated). Of these, nearly half of the DEPs were related to amino acid metabolism. Histidine, lysine and cysteine are considered antioxidant amino acids due to their ability to react more readily with -OH and -SH to achieve antioxidant capacity, and tyrosine is one of the aromatic amino acids used in the synthesis of polyphenols, which protects tissues from oxidative damage by eliminating free radical activity^31–33^. These results suggested that the plants were irreversibly damaged as a result of their downregulation in expression. In addition, there were three DEPs that consistently responded in different time periods of drought stress treatment: Fe2OG dioxygenase domain-containing protein (A0A2N9EQL5) expression was downregulated, indicating that iron ions were continuously released due to environmental instability in plants during the process of adversity stress^34^. Phosphatidylserine decarboxylase proenzyme 2 (PSD2 A0A7N2L1J3) and acyl-coenzyme A oxidase (A0A2N9I7S6) were both upregulated, performing glycerophospholipid metabolism and fatty acid metabolism, respectively. Lipid metabolism provides as much energy as possible to the plant.

### Carbohydrate metabolism-related proteins

Carbohydrates are the main products of photosynthesis in plants, of which nonstructural carbohydrates (NSCs) are important reactants involved in plant life activities and participate in their metabolic processes.NSCs include substances such as soluble sugars and starch, which are important sources of energy during plant growth^29^.

Adverse stress leads to a decrease in photosynthesis, which reduces carbohydrate synthesis and inhibits energy production. In this study, proteomic analysis indicated that 18 DEPs identified in the drought-stressed 2-week leaves, relative to the control, were found to be involved in the regulation of carbohydrate metabolism, of which 16 related proteins showed upregulated expression. Aldedh domain-containing protein (A0A7N2LAP4) and aldehyde dehydrogenase (NAD( +)) (A0A7N2MU33) are substances involved in more pathways in carbohydrate metabolism, and both participate in the processes of pyruvate metabolism, ascorbate and aldarate metabolism, and glycolysis/gluconeogenesis. Their upregulation plays an indelible regulatory role in providing energy for carbohydrate metabolism during the drought suffered by the plant. In addition, a total of seven DEPs were added to the response process of yruvate metabolism, revealing that pyruvate metabolism was also a major substance to resist the drought environment at 2 weeks of drought stress in *C. fissa*. Pyruvate metabolism is the ultimate product from glucose undergoing glycolysis, which is not only oxidized to CO_2_ and H_2_O to complete the oxidative energy supply process but also has a pivotal role in nutrient metabolic linkages by participating in lipid metabolism and amino acid metabolism^6,35^. In addition, pyruvate can induce an increase in the level of ROS in guard cells, which further promotes the closure of stomata and to a certain extent could affect the response ability of plants to drought stress, as described by Wang Mei et al.^36^.

A total of 23 upregulated and 8 downregulated DEPs associated with carbohydrate metabolism were identified in the plant samples treated for 4 weeks. Compared to leaves stressed for 2 weeks, new additions to the pathway of upregulated proteins included the citrate cycle (TCA cycle) and the butanoate metabolism pathway. The TCA cycle is an energy source for plant cells and can provide more energy than glycolysis^37^. Phosphoenolpyruvate carboxykinase (ATP) (A0A7N2N6D6), ATP citrate synthase (A0A2N9IQ08), and oxoglutarate dehydrogenase (succinyl-transferring) (A0A7N2M4C9, A0A7N2MEN2) are involved in the TCA cycle. Phosphoenolpyruvate carboxykinase (ATP) (A0A7N2N6D6), Pyruvate kinase (A0A7N2M5S2, A0A7N2KK32) and three other DEPs (A0A2N9HDG0, A0A7N2KW04, A0A2N9G7S3) are involved in the glycolytic process, and the upregulated expression of these proteins enhances the efficiency of CO_2_ conversion in plants to provide energy to the cells. As analyzed by Sperdouli et al., SS is the main substance involved in carbohydrate metabolism in *A. thaliana* under drought conditions, which not only provides energy but also increases protein stability by reducing the cellular water potential and boosting the water absorption and retention capacity of plant leaves^38^.

### Antioxidant-related proteins

When exposed to adverse conditions, ROS levels in plants increase substantially, causing damage to cellular structures. At this time, the cells induce oxidative stress reactions and perform a complex series of antioxidant protection systems to scavenge excess ROS. Antioxidant systems for ROS elimination are classified into enzymatic and nonenzymatic systems^4,39^. Antioxidant enzymes primarily consist of substances such as SOD, CAT, POD, APX, and GPX, and nonantioxidant enzyme systems mainly include substances such as GSH, AsA, and flavonoids^21,39,40^.

The proteomics data analysis in this study resulted in the identification of 4 antioxidant-related DEPs due to ROS changes in the leaves of drought-stressed plants for 2 weeks. The expression of catalase (A0A2N9HF74, A0A7N2KT83) was upregulated, and the expression of peroxidase (A0A7N2KLJ7, A0A7N2QX64) was downregulated. ROS in plants contain superoxide radicals (O_2_−), hydrogen peroxide (H_2_O_2_), hydroxyl radicals (OH-), and singlet oxygen (^1^O_2_)^20^. SOD consumes excess O^2-^ by performing disproportionation reactions as a first line of defense against ROS-induced damage. CAT can remove H_2_O_2_, a product of fatty acid β-oxidation, photorespiration, etc., into O_2_ and H_2_O; POD assists in purging the H_2_O_2_ produced by the SOD disproportionation reaction, so CAT and POD are the second line of defense in eliminating ROS^20,21^. Phenylpropanoids are necessary for lignin synthesis, which is one of the components of plant cell walls. A higher phenylpropanoid content can contribute to the thickening of cell walls and thus prevent water loss. In the leaves treated for 2 weeks, CAT was upregulated, while POD was downregulated, indicating that CAT performs an important function in scavenging ROS, while POD does not function significantly in adaptation to drought stress. However, the possibility that POD expression in *C. fissa* occurs in other parts (e.g., roots, stems) and less in leaves cannot be excluded. Furthermore, the downregulation of POD expression resulted in the weakening of phenylpropanoid biosynthesis, so it can be speculated that the thinning of the cell wall caused the *C. fissa* leaves to be prone to water loss and curl up^41^ (which is also consistent with the growth state of the sallow cone observed in the experiment).

In the leaves studied for 4 weeks in the experimental treatment, a total of 33 DEPs were identified, including those with superoxide dismutase (A0A7N2L3W0), glutathione peroxidase (A0A7N2N7S0), peroxidase (A0A2N9J2M2, A0A7N2KLJ7, A0A7N2M483, A0A7N2MGN0, A0A7N2QX64) and multiple SHSP domain-containing proteins. With increasing drought stress, SOD (A0A7N2L3W0) and GPX (A0A7N2N7S0) protein expression in leaves was downregulated, resulting in the inhibition of glutathione metabolism and phenylpropanoid biosynthesis. The expression of proteins synthesized by flavonoids was upregulated at this time. Additionally, HSPs are stimulated by drought stress to produce a stress response. Several reports have shown that HSPs act as molecular chaperones under stress conditions, with functions such as maintaining cellular function, resisting oxidative damage, and assisting erroneous protein degradation, and can play a stress-protective role^1,42–44^. The above result indicates that in severe drought, the resistance of the plant decreases and the antioxidant enzyme system almost fails, while the flavonoid content increases greatly to play a certain role in the plant coping with drought.

### Signal transduction-related proteins

When plant organs sense external stimuli, they open the corresponding signaling channels through "signal perception—signal transduction—signal response—activation of physiological/metabolic response". First, plant cells perceive external stimuli through receptor cells located on the cell membrane, which transmit the irritation to secondary messengers (e.g., Ca^2+^, ROS, phosphatidylinositol, sugar and NO), then the secondary messenger is transmitted to the site of action of the receptor cell, and finally, the specific receptor induces the expression of the relevant gene to adapt to the external environment^45^.

In this study, proteomic analysis showed that 6 DEPs identified in the drought-stressed 2-week leaves were found to be involved in the regulation of signaling (5 upregulated and 1 downregulated). In exposure to drought stress, high amounts of ROS are simultaneously responsible for signaling, and they act as secondary messengers/signaling molecules in the signaling process to transmit external environmental signals to the cell interior through redox reactions, thereby activating the MAPK signaling pathway^46,47^. MAPK is an important transmitter of signals from the cell surface to the interior of the nucleus, and the basic composition of its pathway is a conserved three-tier kinase pattern, including MAPK kinase kinase (MKKK), MAPK kinase (MKK), and MAPK, which are activated sequentially by phosphorylation and together regulate a variety of physiological processes such as cell growth, differentiation, and adaptation to environmental stress^48,49^. The phosphatidylinositol signaling system, also known as the dual messenger system, is a signaling pathway that converts extracellular signals into intracellular signals via phospholipase C. The cAMP signaling pathway (cyclic nucleotide system) is able to affect cellular metabolism and cellular behavior by activating cAMP-dependent protein kinase A (PKA) to phosphorylate downstream target proteins. PI3K is one of the central mediators of phospholipid conversion and induces multiple signaling pathways engaged in regulating cellular functions, including Akt (protein kinase B), phosphatidylinositol-dependent protein kinase 1 (PDKI), and the mTOR complex. PI3K-Akt and mTORC1 signaling can promote glycolysis, mitochondrial biogenesis, and fatty acid synthesis^50,51^.

A total of 17 DEPs (16 upregulated and 1 downregulated) were identified that mapped to signaling in the leaves treated with 4 weeks of stress. Among them, five HATPase_c domain-containing proteins (A0A7N2RAC2, A0A2N9ISF4, A0A7N2MGW7, A0A7N2R8J8, A0A7N2LC66) are involved in the PI3K-Akt signaling pathway. Three phospholipase D proteins (A0A2N9EIQ6, A0A7N2LQ78, A0A7N2R4M5) are implicated in the cAMP signaling pathway, phospholipase D signaling pathway, Ras signaling pathway, and sphingolipid signaling pathway. It is evident that both of these types of proteins are key proteins that transduce signals in *C. fissa* during severe drought at this time and can be further investigated.

## Materials and methods

### Plant materials

*C. fissa* seedlings were obtained from the state-owned Dananshan Forestry Farm in Zhaoqing City, Guangdong Province, China (111°54′15″ E, 23°47′31″N). Thirty plants of uniform growth were transplanted into pots 24.5 cm in diameter and 26.5 cm in height in May 2021. Normal maintenance management of the seedlings was performed for 30 days. After the seedlings had established, we selected 15 pots of well-grown seedlings, divided them into five groups of 3 pots each, and place them in a greenhouse and set the temperature to 26 °C and humidity to 60% to ensure environmental stability for the growth of *C. fissa*.

### Stress treatments

The plants were irrigated sufficiently one day before the start of the experiment. One group was the control group (CK group), which was watered quantitatively every 3 days after the start of the stress to maintain its normal growth^52,53^. The other four groups were the experimental drought groups, where the water supply was stopped for 1, 2, 3, and 4 weeks respectively to ensure drought conditions, photosynthesis was measured, and samples were collected on the last day when the water supply was stopped for each group. Samples were collected from 2:30 to 3:00 p.m., from top to bottom of the 3rd to 5th leaves. After treatment, leaves were wrapped separately in precooled 10 ml centrifuge tubes, placed immediately in liquid nitrogen, brought back to the laboratory, and stored at − 80 °C for further analysis. These samples will be used for both physiological experiments on the leaves of *C. fissa* and protein profiling experiments.

### Physiological measurements

The photosynthetic index, antioxidant enzyme activity, MDA level and osmolyte contents were measured in each treatment group. The P_*n*_, T_*r*_, C_*i*_, and G_*s*_ of the plants were measured using a Li-6800 photosynthesizer (USA) on a sunny day, and the light intensity was set at 1200 μmol/(m^2^$\cdot$s). SOD activity was determined by colorimetric analysis of the water-soluble formazan dye catalyzed by WST-1 at 450 nm (WST-1). POD activity was derived from its enzymatic activity by measuring the change in absorbance at 420 nm of its catalytic H_2_O_2_. CAT activity was calculated by tracking the reduction of H_2_O_2_ using ammonium molybdate. The MDA content was measured using a modified thiobarbituric acid (TBA) method^54^. The Pro content was determined by the acidic ninhydrin method at the maximum absorption peak at 515 nm^55^. The SS content was obtained by monitoring colored substances produced by sugar and anthrone at 630 nm. The SP content was calculated by measuring the absorbance using Komas Brilliant Blue solution. For the determination of physiological and biochemical indexes of plants, each index was repeated three times.

### Protein extraction and concentration determination

The protein concentration was determined by the bicinchoninic acid (BCA) method. A clean 96-well plate was prepared, and different volumes of bovine serum albumin (BSA) standard protein solution was added to wells as follows: 0, 1, 2, 4, 8, 12, 16, and 20 µL. Then, the corresponding volume of ultra-pure water was added to each well to bring the volume to 20 µL. Two microliters of protein solution was added to a 96-well plate with triplicate wells for each sample, and the volume was brought to 20 µL. Then 200 µL of preconfigured working reagent was added to each well, and the mixture was incubated for 30 min at 37 °C. The absorbance value (wavelength 562 nm) was determined by an enzyme labeling instrument. Then, by calculating the standard curve according to the known concentration and absorbance value of the standard protein solution and substituting the absorbance value of the sample to be measured, the protein concentration value was calculated.

### Protein digestion and TMT labeling

According to the measured protein concentration, the same quality protein was taken from each sample, and the samples of the CK, DR2w and DR4w groups were diluted with lysis solution to the same concentration and volume. Dithiothreitol (DTT) was added to the above protein solution to a final DTT concentration of approximately 5 mM, mixed well and incubated at 55°C for 30 min. After cooling to room temperature, the corresponding volume of iodoacetamide was added so that the final concentration was approximately 10 mm, and the mixture was placed in the dark for 15 min. Then, 6 times the volume of precooled acetone was added to the above system to precipitate the protein, which was placed at -20°C for four hours. After precipitation, the sample was removed and centrifuged at 8000 × *g* for 10 min at 4 °C to collect the precipitate, and volatilized acetone was added for 2–3 min. According to the amount of protein, the corresponding volume of enzymolysis diluent (protein: enzyme = 50:1 (m/m), 100 µg of protein with 2 µg of enzyme) was added to redissolve the protein precipitate, and then the solutions were incubated for digestion at 37 °C for 12 h. Finally, the samples were lyophilized or evaporated after enzymolysis.

To perform peptide labeling, the lyophilized samples were resuspended in 50 μL of 100 mM triethanolamide buffered saline (TEAB), vortexed and then transferred into new 1.5 mL tubes for the labeling reaction. Twenty microliters of anhydrous acetonitrile was added to the TMT reagent vial at room temperature. The centrifuged reagents were dissolved for 5 min and mixed for centrifugation, and this step was repeated once. Then, 10 μL of the TMT pro label reagent was added to each sample for mixing according to the labeling information table (Table 3). The tubes were incubated at room temperature for 1 h. Finally, 5 μL of 5% hydroxylamine was added to each sample and incubated for 15 min to terminate the reaction. The labeled peptide solutions were lyophilized and stored at − 80 °C.

**Table 3:** Peptide labeling information table.

Marker number	126	127N	127C	128N	128C	129N	129C	130N	130C
Sample number	CK-1	CK-2	CK-3	DR2w-1	DR2w-2	DR2w-3	DR4w-1	DR4w-2	DR4w-3

### LC‒MS/MS analysis and database search

Before mass spectrometry (MS) analysis, the tryptic peptides were fractionated on an 1100 HPLC System (Agilent) using an Agilent Zorbax Extend RP column (5 μm, 150 mm × 2.1 mm). Mobile phases A (2% acetonitrile in HPLC water) and B (98% acetonitrile in HPLC water) were used for the RP gradient. The solvent gradient for mobile phase A was set. Tryptic peptides were separated at a fluent flow rate of 300 μL/min and monitored at 210 and 280 nm. Samples were collected for 8–60 min, and the eluent was collected in centrifugal tubes 1–15 every minute in turn. Samples were recycled in this order until the end of the gradient. The separated peptides were lyophilized for mass spectrometry.

All analyses were performed by an EASY-nLC 1200 UPLC system (Thermo Fisher Scientific, Waltham, MA, USA). The flow rate was 300 nL/min, and the linear gradient was 75 min (0 ~ 50 min, 5–28% B; 50 ~ 60 min, 28–42% B; 60 ~ 65 min, 42–90% B; 65 ~ 75 min, 90% B. Mobile phase A = 0.1% FA in water and B = 80% ACN/0.1% FA in water). The full MS scan range was 300–1500 m/z, with a mass resolution of 60,000, and the AGC target value was 3e6. The ten most intense peaks in MS were fragmented with high-energy collisional dissociation (HCD) with an NCE of 32. MS/MS spectra were obtained with a resolution of 45,000, an AGC target of 2e5, and a max injection time of 80 ms. The Q-E dynamic exclusion was set for 30.0 s and run under positive mode.

### Statistical analysis

All physiological index data were analyzed using SPSS 22.0 (SPSS Institute, Inc., USA), and for their relative quantification, protein expression abundance was set to determine the statistical significance of the difference and accurately identify the DEPs induced by drought stress. When the protein difference multiple was > 1.5 and its *P*-value was < 0.05, it was considered an upregulated protein; when the difference multiple was < 0.66 and the *P*-value was < 0.05, the protein was designated as downregulated. Proteins identified in different groups were used to generate a heat map using TBtools v1.112 (https://github.com/CJ-Chen/TBtools/tags) based on their expression level differences. The functions of all identified proteins were determined by GO analysis in the UniProt database (http://www.uniprot.org), and proteins were classified according to their main functions. We used the string-DB (http://string-db.org/) protein interaction database (selecting *Arabidopsis thaliana*) to analyze the interaction of the compared and DEPs.

### Ethics approval and consent to participate

The experimental research and field studies on plants or seeds in this work comply with the IUCN Policy Statement on Research Involving Species at Risk of Extinction and the Convention on the Trade in Endangered Species of Wild Fauna and Flora. Experimental research in this study on plants in this study, including the collection of plant material, complies with relevant institutional, national, and international guidelines and legislation. The permission of *Castanopsis fissa* plant seedlings collection was obtained, and the plant specimens are located in the herbarium at the School of Life Sciences, South China Normal University, with voucher number SN SN012073.

## Conclusion

In this study, we comprehensively compared and analyzed the physiological and proteomic changes of *C. fissa* plants subjected to 2-week and 4-week drought stress treatments with those of the control group. Our results showed that under drought stress, the degree of membrane lipid peroxidation in *C. fissa* significantly increased, and the accumulation of osmotic adjustment substances affected its normal physiological metabolism. However, *C. fissa* was not affected by non-stomatal limitations, and the increased activity of antioxidant enzymes indicated that photosynthesis and antioxidant enzymes regulated its drought resistance to a certain extent. The expression levels of DEPs identified using TMT technology involved in photosynthesis, antioxidants and defense, carbohydrate metabolism, signal transduction, and protein synthesis and degradation were generally upregulated, while those involved in energy metabolism were generally downregulated. In conclusion, through the analysis of the physiological responses and proteome of *C. fissa*, we have identified key pathways and proteins involved in drought stress, providing a basis for the study of its drought resistance mechanism, as well as a foundation for the study of drought tolerance in other plants.

## Supplementary Information

Supplementary Table S1.

Supplementary Table S2.

Supplementary Table S3.

## Data Availability

The mass spectrometry proteomics data have been deposited to the ProteomeXchange Consortium via the PRIDE partner repository with the dataset identifier PXD038837.
